# Supplementary material for: The genome sequence of the turbot, Scophthalmus maximus (Linnaeus, 1758) (Pleuronectiformes: Scophthalmidae)
Source: Wellcome Open Res. 2025 Oct 21;10:592. [Version 1] doi: 10.12688/wellcomeopenres.25070.1 (PMC12775657; doi:10.12688/wellcomeopenres.25070.1)
Supplement: Peer review history

# Reviewer report: Reviewer response for version 1

**DOI:** 10.21956/wellcomeopenres.27637.r141840

**Published:** 2026-01-06

**Authors:** Jessica Winn

**Affiliations:** 1 Stellenbosch University, Stellenbosch, South Africa

**Summary**

 This manuscript presents a high-quality chromosome-level genome assembly for the turbot ( *Scophthalmus maximus*), a commercially important European flatfish species. The assembly was generated using PacBio HiFi long reads (20.53 Gb, ~41× coverage) scaffolded with Hi-C data (177.55 Gb), resulting in 550.28 Mb genome with 22 chromosomal pseudomolecules representing 99.74% of the assembly. The work achieves excellent quality metrics (QV 54.8, 99.0% BUSCO completeness) and meets Earth BioGenome Project reference standards (6.C.Q53). This assembly was produced as part of the Darwin Tree of Life Project.

**Strengths**

 The manuscript demonstrates exemplary best practices in several areas:

- Thorough documentation: Comprehensive software table (Table 5) with versions and sources
- Reproducibility: Protocol.io links, GitLab curation documentation, and public data availability
- Quality metrics: Thorough QC including BUSCO, k-mer completeness, QV scores, and EBP benchmarking
- Transparency: Detailed curation steps quantified (23 breaks, 41 joins, 3 haplotypic duplications removed)
- High-quality assembly: Excellent contiguity (N50 26.14 Mb) and completeness
- Clear presentation: Well-organized tables and informative figures
- Ethical compliance: Thorough legal/governance section and sampling protocols

**Key issues**

 1. Scophthalmidae species count

 Background (paragraph 1): "The Scophthalmidae family consists of at least 38 identified species of flatfish"

 This statement is misleading. While Fricke (2020) does list 38 available species names for Scophthalmidae, only 9 are taxonomically valid species distributed across 4 genera. The remaining names likely represent synonyms, subspecies, or historically recognized taxa no longer considered valid. The current wording incorrectly suggests 38 distinct valid species exist. Reword to accurately reflect taxonomic validity and provide some more phylogenetic context to prevent confusion about the family’s diversity.

 2. Incomplete results reporting

 Genome sequence report: Methods mentions ASCC decontamination pipeline was run, but Results never reports the outcome.

 Add one sentence stating either that no contamination was detected during ASCC screening or state how many contaminant scaffolds and bases were identified and removed.

**Minor suggestions**

*Background section*

 1. Missing punctuation

 Paragraph 1: "in the northeast Atlantic Ocean the Black Sea…"

 Add comma: "in the northeast Atlantic Ocean*,* the Black Sea…"

 2. Vague language

 Paragraph 1: "holds global significance" is imprecise.

 Suggestion: "is of significant economic and ecological importance" or "is commercially and ecologically important"

 3. Awkward phrasing

 Paragraph 3: "Initially selected for aquaculture in the 1970s due to its commercial value in the United Kingdom and France, turbot aquaculture began in Scotland and has since expanded..."

 Suggestion: "Turbot was initially selected for aquaculture in Scotland in the 1970s due to its high commercial value in European markets, and production has since expanded to other European countries and China."

*Methods section*

 4. Table 5 reference placement

 Table 5 (comprehensive software versions) is mentioned only in the Data availability statement but should be referenced in the Methods.

 Suggestion: Add to first paragraph of Methods: "All software tools and versions used in this study are listed in Table 5". Otherwise move this table into the Methods section.

 5. RNA-seq fragment size clarity

 "Libraries were size-selected to produce fragments between 100-300 bp" seems quite small.

 Clarify whether this refers to cDNA library insert size or final library size including adapters.

 6. Hi-C pooling specificity

 "Normalised libraries were quantified again and equimolar and/or weighted 2.8 nM pools"

 This sentence is unclear. Specify which approach was used (equimolar OR weighted, not both). If different pools used different strategies, this should be specified.

*Genome assembly report section:*

 7. RNA-seq purpose

 Sequence data: "RNA sequencing data were also generated and are available in public sequence repositories."

 Suggestion: Consider adding a forward reference: "RNA sequencing data were generated for genome annotation and are available in public repositories (see Data Availability)."

 8. Hi-C quality metrics

 Sequence data: Hi-C data volume is reported but not quality metrics.

 Consider adding the percentage of valid pairs and long-range contacts with the read number to validate scaffolding quality if available.

 9. Gap characterization

 Assembly statistics: "805 gaps" is mentioned but not explained.

 Add one sentence clarifying these are Hi-C scaffold gaps (inter-contig junctions) and provide average estimated size if available, e.g.: 'The 805 gaps represent junctions between contigs within Hi-C scaffolds, with an average estimated size of ~X bp.'"

 10. Curation impact clarity

 Methods quantify curation (23 breaks, 41 joins, 3 haplotypic duplications) and states "15.6% reduction" but the genome assembly report doesn't connect these.

 Suggestion: Expand on this in the assembly report. "Manual curation reduced the initial scaffold count from X to 107 (15.6% reduction) through 23 breaks, 41 joins, and removal of three haplotypic duplications."

**Conclusion**

 This is a high-quality genome paper that demonstrates exemplary documentation and reproducibility practices. The assembly quality is excellent and meets international standards. With correction of some minor issues, the manuscript will be scientifically sound and ready to be indexed.

Are sufficient details of methods and materials provided to allow replication by others?

Partly

Is the rationale for creating the dataset(s) clearly described?

Yes

Are the datasets clearly presented in a useable and accessible format?

Yes

Are the protocols appropriate and is the work technically sound?

Partly

Reviewer Expertise:

My research expertise is in phylogenomics, population genomics, and molecular ecology, with experience in bioinformatics pipelines and genome analysis. While I have assembled mitogenomes and worked with genomic data, I have not personally conducted whole-genome assembly using PacBio HiFi reads. I evaluated this manuscript based on my genomic data analysis experience, comparison with published genome assemblies, and review of relevant literature, though I may lack some technical nuances specific to long-read assembly workflows.

I confirm that I have read this submission and believe that I have an appropriate level of expertise to confirm that it is of an acceptable scientific standard.

# Reviewer report: Reviewer response for version 1

**DOI:** 10.21956/wellcomeopenres.27637.r139494

**Published:** 2025-11-24

**Authors:** Junrou Huang

**Affiliations:** 1 Sun Yat-sen University, Guangzhou, China

This manuscript presents a high-quality, chromosome-level genome assembly for the turbot, generated under the Darwin Tree of Life project. The assembly demonstrates excellent quality, with 99.74% of the sequence anchored into 22 chromosomes, high BUSCO completeness, and robust quality scores. The integration of PacBio HiFi, Hi-C, and RNA-seq data, followed by manual curation, has resulted in a reference-grade genome that meets the standards of the Earth BioGenome Project. The data are comprehensively documented and made readily available to the community. This resource will be invaluable for research in areas such as aquaculture, comparative genomics, and conservation biology.

 I recommend indexing of this manuscript.

 The study is technically sound and the data represent a significant contribution. However, the Introduction would be strengthened by explicitly stating the rationale for generating a new genome assembly, given the prior existence of a chromosome-level assembly (GCF_022379125.1). A concise justification would better contextualize the necessity and added value of this work.

Are sufficient details of methods and materials provided to allow replication by others?

Yes

Is the rationale for creating the dataset(s) clearly described?

Partly

Are the datasets clearly presented in a useable and accessible format?

Yes

Are the protocols appropriate and is the work technically sound?

Yes

Reviewer Expertise:

Genomics, Comparative genomics, Single-cell transcriptomics

I confirm that I have read this submission and believe that I have an appropriate level of expertise to confirm that it is of an acceptable scientific standard.
